# Bioinspired Origami Morphing Limbs for Amphibious Robot Locomotion

**DOI:** 10.3390/biomimetics11070502

**Published:** 2026-07-17

**Authors:** Yuxuan Li, Siyu Mei, Rensong Yin, Chong Liu, Hui Chen

**Affiliations:** 1 Center for Mechanics Plus under Extreme Environments, School of Mechanics and Engineering Science, Ningbo University, Ningbo 315211, China; 2311090143@nbu.edu.cn (Y.L.); 2411090194@nbu.edu.cn (S.M.); 2211090042@nbu.edu.cn (R.Y.); 2401090039@nbu.edu.cn (C.L.); 2 Piezoelectric Device Laboratory, School of Mechanics and Engineering Science, Ningbo University, Ningbo 315211, China

**Keywords:** amphibious robot, bioinspired robotics, origami-inspired mechanism, morphing limb, Yoshimura pattern

## Abstract

Amphibious robots must reconcile two distinct mechanical requirements within a compact locomotion architecture. Terrestrial operation requires limb structures with sufficient load-bearing capacity, contact stability, and bending resistance, whereas aquatic operation benefits from a larger projected area for drag-based thrust generation. Conventional amphibious platforms often address these requirements by combining separate land and water propulsion modules, which increases structural redundancy, system mass, and hydrodynamic resistance. To reduce this conflict at the structural level, this study proposes a bioinspired origami morphing limb based on a modified Yoshimura pattern. The limb transforms between a closed cylindrical configuration for terrestrial support and an unfolded planar configuration for aquatic paddling. A vertex-splitting topology and thick-panel geometric constraints are introduced to suppress the bifurcation instability associated with the zero-thickness Yoshimura vertex, thereby obtaining a deterministic single-degree-of-freedom folding path suitable for robotic actuation. A screw-theory-based kinematic model is established to relate the active driving angle to the passive folding angle, and geometric parameter analysis is used to connect the folding state with load-bearing and paddling morphologies. A quadruped amphibious robot prototype is fabricated using rigid polylactic acid panels and flexible thermoplastic polyurethane hinges. Prototype-level observations qualitatively demonstrate reversible transformation within the tested operating range and show walking, crawling, rolling, water-entry, and underwater locomotion modes.

## 1. Introduction

Amphibious animals provide examples of locomotion systems that operate across discontinuous physical environments. When moving on land, their limbs interact with a solid substrate and must support body weight, generate traction, and maintain postural stability. When moving in water, the same appendages interact with a fluid medium and must generate thrust through drag or lift. These requirements are not identical and can even be conflicting. A slender or compact limb is beneficial for reducing resistance and improving support efficiency on land, whereas an expanded surface is preferred for increasing thrust during aquatic paddling. This morphological adaptation principle is relevant to amphibious robotics, where a single robotic platform is expected to move across shorelines, wetlands, shallow water, and land–water transition zones. Amphibious robots have therefore been investigated for coastal exploration, ecological monitoring, inspection, disaster response, and operations in unstructured environments [1,2,3,4].

Despite extensive progress, the design of compact and mechanically efficient amphibious locomotion systems remains difficult. Many existing amphibious robots adopt an additive configuration, in which wheels, tracks, legs, fins, flippers, or propellers are combined on the same body to satisfy different locomotion modes [5,6,7,8]. This strategy improves functional coverage but introduces structural redundancy. Components designed for one environment may become inactive mass in another environment. For example, propellers and fins may increase drag or occupy space during terrestrial locomotion, while wheels or rigid legs may reduce hydrodynamic efficiency during swimming. Such redundancy can also increase the number of actuators, complicate the transmission system, and raise the demand on control coordination. To reduce mechanical redundancy, recent studies have explored adaptive, soft, and morphing structures for amphibious locomotion [9,10,11,12,13,14,15]. These approaches use structural deformation or stiffness variation to adapt the robot to different media. Soft structures can conform to terrain and generate fluidic motion, but their low stiffness may limit load-bearing capacity under terrestrial contact. Rigid-link mechanisms provide higher support capability, but they often require multiple joints, actuators, and locking components to achieve shape transformation. Therefore, a design method that combines structural stiffness, lightweight construction, and low-actuation reconfigurability is required for amphibious robots.

Origami-inspired mechanisms provide a feasible route for this requirement. Origami structures can generate large geometric transformations through folding, while maintaining compactness and low structural mass [16,17,18,19,20,21,22,23]. In addition, origami kinematics can often be described by a limited number of generalized coordinates, which facilitates mechanical actuation and mode switching. For robotic applications, however, the ideal zero-thickness origami model must be converted into a finite-thickness structure. Thick-panel origami provides the geometric basis for fabricating load-bearing foldable structures using rigid panels and mechanical or compliant hinges [24,25]. The selection of the crease pattern is therefore a key design factor. The Miura-ori pattern has high planar deployability, but it does not naturally form a closed tubular cross-section for load-bearing support. The Kresling pattern has a cylindrical topology, but axial folding is usually accompanied by pronounced torsion, which increases the complexity of actuation and foot orientation control. The Yoshimura pattern is suitable for this study because it can form a cylindrical shell-like morphology while also allowing expansion toward a planar state [26,27]. This geometric characteristic matches the required transformation between a terrestrial column and an aquatic paddle.

To clarify the positioning of the proposed limb relative to representative amphibious and morphing robots, Table 1 summarizes several recent design routes. Existing amphibious platforms have achieved impressive land–water mobility using paddle-wheel mechanisms, adaptive morphing limbs, soft pneumatic bodies, or small-scale origami actuation [5,10,11,12,15,23]. However, their design objectives, scales, actuation principles, and validation methods differ substantially. Therefore, a direct quantitative performance ranking is not appropriate without controlled experiments under identical conditions. The present work is positioned as a proof-of-concept structural design that focuses on reducing limb-level functional redundancy by assigning terrestrial support and aquatic paddling to the same reconfigurable origami limb.

This paper presents a bioinspired origami morphing limb for amphibious robots. The main concept is to integrate two locomotion functions into one reconfigurable structural unit: a closed cylindrical state for terrestrial load-bearing and an unfolded planar state for aquatic propulsion. The design is based on a modified Yoshimura pattern. To improve motion determinacy, vertex splitting and thick-panel constraints are introduced to suppress the bifurcation behavior observed in the zero-thickness configuration. A screw-theory-based kinematic model is established to derive the relationship between the active driving crease and passive folding motion. The geometric transformation is analyzed to connect the folding state with the functional morphology of the limb. Finally, a quadruped prototype is fabricated and tested to qualitatively demonstrate morphological transformation and multimodal locomotion. The contributions of this study are summarized as follows. First, a morphing origami limb is proposed to combine land support and water paddling within the same mechanical structure. Second, a modified Yoshimura topology is developed to obtain a deterministic folding path through vertex splitting and finite-thickness geometric constraints. Third, a kinematic model is established using screw theory to describe the relationship between actuation and passive folding. Fourth, a physical amphibious robot prototype is implemented and qualitatively evaluated in walking, crawling, rolling, water-entry, and swimming scenarios.

## 2. Bioinspired Origami Limb Design and Kinematic Analysis

**Figure 1:**
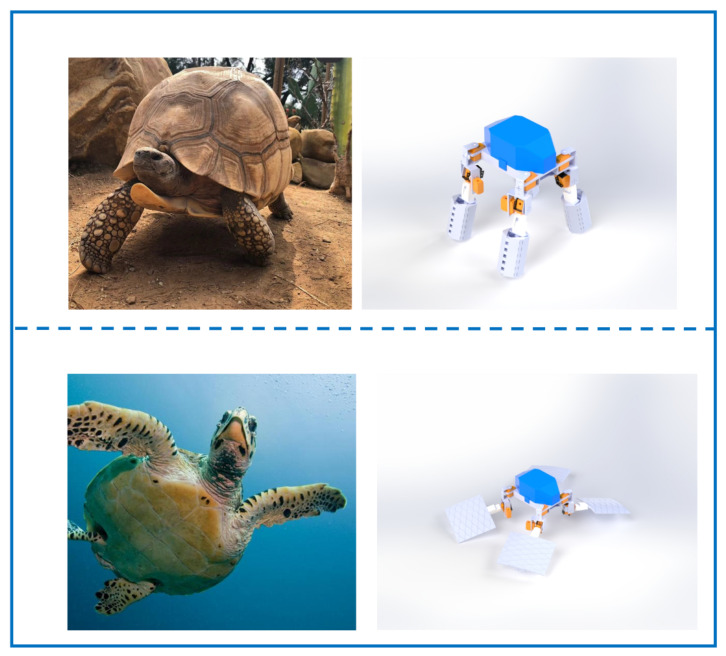
Biological inspiration for the morphing limb. A tortoise using robust columnar limbs for terrestrial load-bearing (**upper** panel). A sea turtle using flat, flipper-like limbs for aquatic propulsion (**bottom** panel).

The proposed limb is motivated by the functional contrast between terrestrial support and aquatic propulsion. Rather than reproducing the anatomy of a specific species, the design abstracts a mechanical principle observed in amphibious animals: a single appendage can change its effective morphology according to the dominant interaction medium. In the terrestrial state, the limb forms a closed cylindrical structure. The closed cross-section increases the second moment of area and improves resistance to lateral bending, which is beneficial for supporting the robot body during stance phases. In the aquatic state, the same structure unfolds into a planar paddle. The increased projected area provides a geometric basis for potential drag-based fluid interaction during the power stroke. The biological analogy used for this functional abstraction is shown in Figure 1. In this study, the bioinspiration is at the functional-morphology level rather than at the anatomical-topology level. The Yoshimura pattern is an engineering origami topology, while the biological analogy is used to motivate the transition between a compact load-bearing limb and an expanded paddle-like limb. The design process starts from the Yoshimura crease pattern and then modifies its local vertex topology to satisfy the reliability requirements of robotic implementation. For an amphibious robot, a repeatable folding path is essential because uncertain branch selection may lead to incomplete deployment, undesired inversion, or loss of support during ground contact. The following subsections therefore describe the topology evolution, the finite-thickness constraint strategy, and the kinematic model used to characterize the deterministic folding path.

### 2.1. Topological Evolution and Singularity Elimination

**Figure 2:**
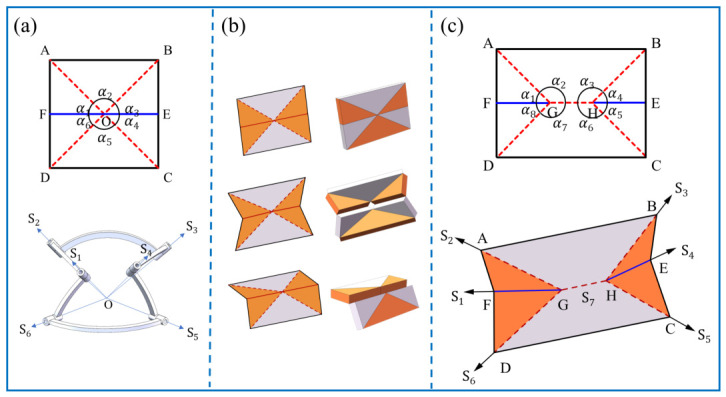
Evolution of the origami mechanism and the principle of deterministic folding. (**a**) The standard single-vertex Yoshimura pattern (zero-thickness model) at the flat singularity. (**b**) Kinematic analysis reveals a bifurcation where the structure can fold into either a cylindrical mode or snap through to an inverted mode. (**c**) The vertex-split topology with a central shared crease. In the zero-thickness idealization, this configuration is kinematically equivalent to a Bricard 6R linkage, retaining the inherent bifurcation instability (singularity persists).

The standard Yoshimura pattern contains a single-vertex, six-crease topology. In an ideal zero-thickness model, this topology offers a large folding ratio and a regular cylindrical morphology. However, the fully deployed flat configuration is a singular state in which the rank of the kinematic constraint matrix decreases. As a result, the mechanism can leave the singular configuration along more than one admissible folding branch. As illustrated in Figure 2a, one branch leads to the desired cylindrical mode, whereas another branch may produce an inverted or snap-through configuration. For a robotic limb subjected to external contact loads, this branch ambiguity is undesirable because it reduces transformation reliability and may trigger local collapse.

**Figure 3:**
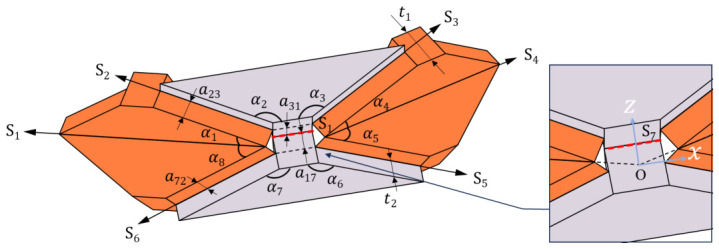
Geometric parameters and coordinate system of the modified thick-panel Yoshimura module. The global coordinate system {S} is established at the geometric center. The proposed thick-panel design. The introduction of panel thickness creates a unilateral geometric constraint. As shown in the zoom-in view, any attempt to fold along the invalid path triggers volumetric interference between adjacent panel edges, physically blocking the bifurcation and enforcing a deterministic folding trajectory along the feasible path.

To reduce this ambiguity, a vertex-splitting strategy is introduced. The original single vertex is replaced by a double-vertex topology with a central shared crease, forming a seven-crease local pattern, as shown in Figure 2b. This topological modification separates the intersecting crease axes and provides a structural basis for finite-thickness fabrication. However, the zero-thickness equivalent of the split topology does not automatically eliminate the singularity. The loop formed by the split vertices and the shared crease can be interpreted as a Bricard 6R linkage, which is also related to two coupled Bennett 4R loops (Figure 2c). Under the zero-thickness assumption, redundant mobility may still exist near the flat configuration, and the folding branch may remain undetermined. The proposed solution therefore relies on the physical implementation of the thick-panel structure. Once a finite panel thickness *t* is introduced, the shared crease is no longer only a mathematical line but a spatial constraint with offset joint axes and finite panel boundaries. The hinge-shifted geometry changes the local compatibility conditions and introduces unilateral contact constraints between adjacent panel edges. As shown in Figure 3, folding along the undesired branch causes geometric interference, whereas folding along the designed branch remains feasible. The invalid branch is therefore mechanically blocked, and the structure follows a deterministic folding route from the planar state to the cylindrical state. This treatment converts the singularity problem of an ideal crease pattern into a constraint-design problem for a manufacturable thick-panel mechanism.

### 2.2. Kinematic Modeling via Screw Theory

Screw theory is used to describe the spatial folding motion of the modified Yoshimura module. Compared with a local coordinate description, the screw-theory formulation represents revolute crease axes and their motions in a unified spatial form, which is convenient for closed-loop compatibility analysis. The idealized zero-thickness equivalent mechanism is modeled as a plane-symmetric spherical 6R closed chain. Each crease is treated as a zero-pitch revolute screw. According to the geometry of the double-vertex pattern, the initial unit twists $\xi_{i(0)}\in R^{6}$ (i=1,2,…,6) for the six joint axes are defined as:

(1) $$\begin{array}{cc} \xi_{1}\left(0\right) & ={\left[-1,0,0;0,0,0\right]}^{T},\\ \xi_{2}\left(0\right) & ={\left[-\cos\alpha ,0,\sin\alpha ;0,0,0\right]}^{T},\\ \xi_{3}\left(0\right) & ={\left[\cos\alpha ,0,\sin\alpha ;0,0,0\right]}^{T},\\ \xi_{4}\left(0\right) & ={\left[1,0,0;0,0,0\right]}^{T},\\ \xi_{5}\left(0\right) & ={\left[\cos\alpha ,0,-\sin\alpha ;0,0,0\right]}^{T},\\ \xi_{6}\left(0\right) & ={\left[-\cos\alpha ,0,-\sin\alpha ;0,0,0\right]}^{T}, \end{array}$$

where α denotes the sector angle of the origami panel. In the spherical equivalent model, the revolute axes intersect at the ideal vertex, and the moment terms of the twists are therefore zero. The zero-thickness spherical 6R model is an idealized kinematic reference rather than a high-precision predictive model of the fabricated thick-panel prototype. In the fabricated PLA–TPU limb, finite panel thickness, hinge offsets, TPU hinge compliance, friction, and manufacturing tolerances can introduce deviations from the idealized angular relationship. Therefore, the model is used in this study to clarify the folding-branch selection and the coupling between active and passive crease angles, rather than to provide a complete high-precision prediction of the prototype motion. Quantitative comparison between measured folding angles and theoretical predictions will be required in future work. The closed-loop kinematic compatibility condition is then expressed using the Product of Exponentials formulation:

(2) $$e^{{\hat{\xi }}_{1}\theta_{1}}e^{{\hat{\xi }}_{2}\theta_{2}}\cdots e^{{\hat{\xi }}_{6}\theta_{6}}=I,$$

where $\theta_{i}$ represents the joint angle of the *i*-th axis. Because the modified module is designed with plane symmetry and is constrained to follow one folding branch, the motion can be reduced to a single generalized input. The angular variables therefore satisfy the following symmetric constraints:

(3) $$\theta_{1}=\theta_{4},\;\theta_{2}=\theta_{3}=\theta_{5}=\theta_{6}.$$

**Figure 4:**
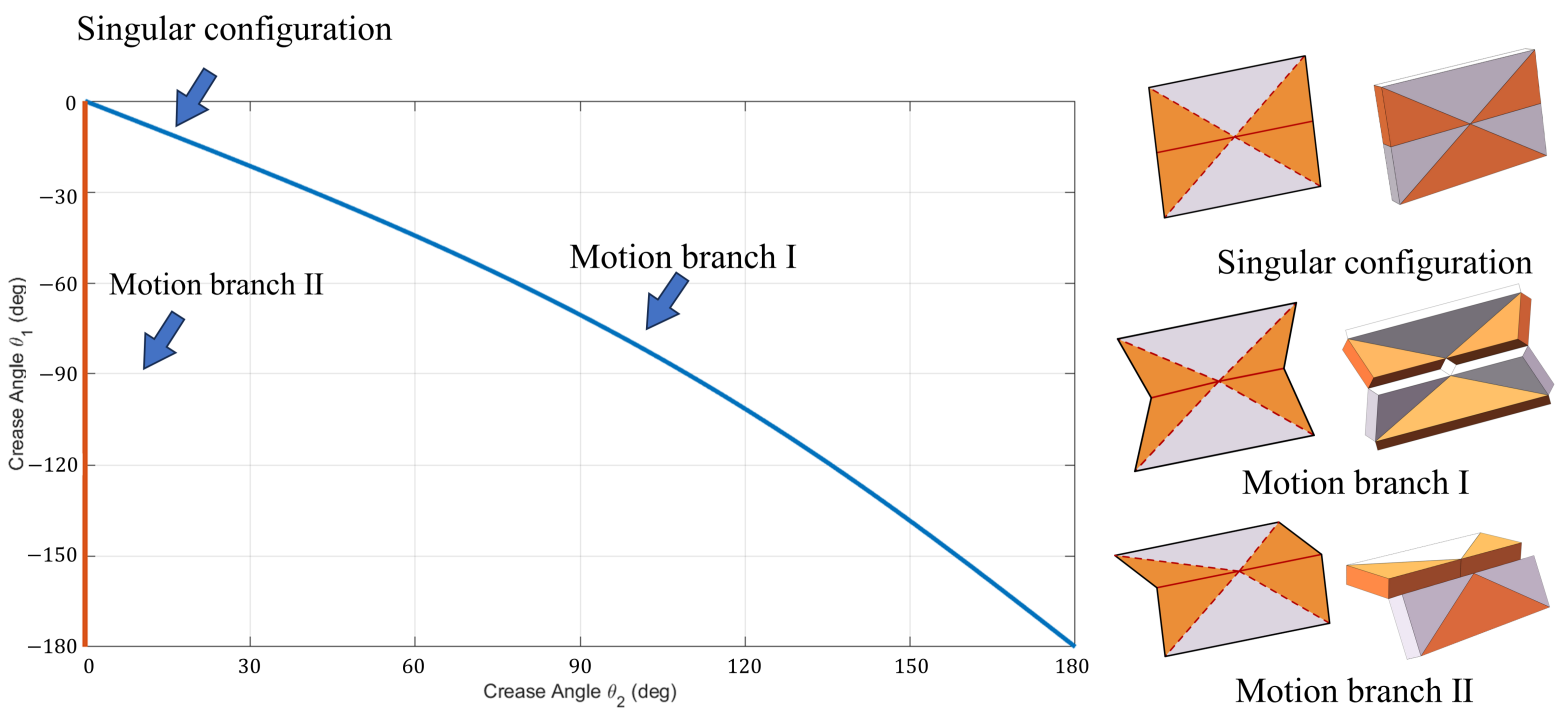
Kinematic bifurcation of a Yoshimura thick-origami mechanism. The mechanism reaches a singular configuration in its fully flat state, where the kinematic path splits into two distinct routes: Motion branch 1 and Motion branch 2, representing different folding behaviors originating from the singularity.

**Figure 5:**
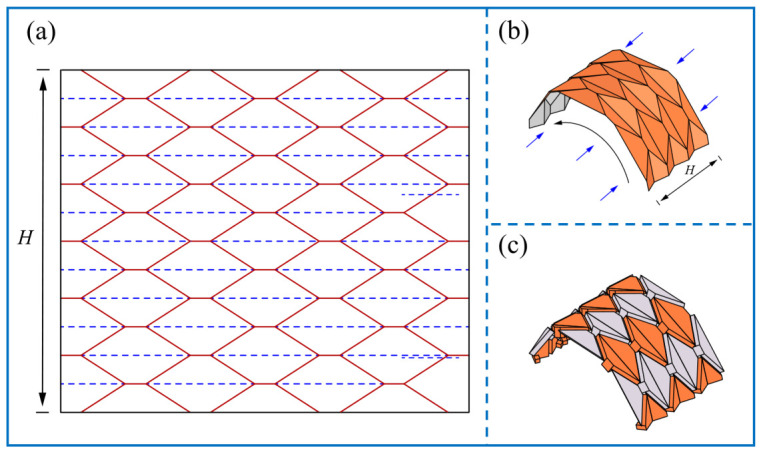
Geometric modeling and deformation principle of the Yoshimura origami pattern. (**a**) The complete tessellated crease pattern composed of 3 rows and 7 columns of trapezoidal units. (**b**) The bending deformation tendency of the zero-thickness model: lateral compression (indicated by blue arrows) induces a macroscopic curvature along the black arc. (**c**) The corresponding thick-panel model, demonstrating identical kinematic evolution and a one-to-one mapping with the zero-thickness counterpart.

By substituting Equations (1) and (3) into the loop-closure equation, the relationship between the active driving angle $\theta_{1}$ and the passive folding angle $\theta_{2}$ is obtained. The resulting closed-form expression is

(4) $$\tan\frac{\theta_{1}}{2}=-\cos\alpha \tan\frac{\theta_{2}}{2},$$

describing the ideal folding relationship of the zero-thickness equivalent module. The corresponding kinematic bifurcation is illustrated in Figure 4, where the fully flat configuration represents a singular state separating the two admissible motion branches. For a fabricated limb, the finite panel thickness and hinge offsets must also satisfy spatial compatibility. The hinge-shift technique is therefore used to convert the spherical mechanism into a spatial closed-loop linkage while preserving the desired one-degree-of-freedom behavior. For the spatial loop formed by the split vertices in Figure 3, the Bennett-linkage compatibility condition can be written as

(5) $$\alpha_{1}+\alpha_{7}=\pi ,\;\frac{\sin\alpha_{1}}{d_{12}}=\frac{\sin\alpha_{7}}{d_{71}},$$

where $d_{ij}$ represents the spatial offset distance between non-intersecting joint axes and is related to the finite panel thickness *t*. These geometric constraints prevent the hinge offsets from either introducing uncontrolled additional mobility or over-constraining the folding path. Consequently, the thick-panel module maintains the coupled axial–radial transformation required for the amphibious limb while remaining compatible with physical fabrication [25]. The tessellated Yoshimura crease network is shown in Figure 5.

## 3. Functional Morphology and Geometric Parameter Selection

The functional performance of the morphing limb is governed by the relationship between the crease-pattern parameters and the resulting three-dimensional morphology. The design must satisfy two competing requirements. In terrestrial mode, the folded configuration should form a closed or nearly closed cylindrical cross-section to provide structural support. In aquatic mode, the unfolded configuration should provide a larger projected area for potential drag-based fluid interaction. These requirements are coupled through the sector angle α, the circumferential module number *N*, the axial layer count *M*, the panel slant height, and the finite-thickness hinge layout. The following analysis connects these geometric parameters with the cylindrical and planar limit states of the limb.

For the prototype design, the basic double-vertex topology is specified by a sector angle of $\alpha =30^{\circ }$, a vertex vertical offset of $d_{v}=10\;\mathrm{mm}$, and a panel slant height of h=30mm. This parameter set was selected to satisfy circumferential closure in the folded state while keeping the unfolded paddle span within the available actuator stroke and prototype envelope. The resulting geometry provides a repeatable transformation between the two functional morphologies without requiring an additional locking mechanism in the tested configuration.

### 3.1. Geometric Definition and Circumferential Closure

In a tessellated Yoshimura structure, the local folding of each module accumulates along both the circumferential and axial directions. For a chain consisting of *n* units, the orientation of the terminal coordinate frame is determined by the cumulative angular deviation of all modules. Let $\xi_{i}$ denote the axis deviation angle of the *i*-th unit’s final coordinate system relative to its initial coordinate system. This angle represents the deterministic orientation change associated with folding and describes the rotation of the limb cross-section during axial compression or extension. Based on the spherical geometric constraints of the double-vertex pattern, the explicit relationship between the deviation angle $\xi_{i}$, the sector angle $\alpha_{i}$, and the folding dihedral angle θ is derived as

(6) $$\cos\xi_{i}=\frac{\sin^{2}\alpha_{i}\cos^{2}\frac{\theta }{2}-\cos^{2}\alpha_{i}}{\sin^{2}\alpha_{i}\cos^{2}\frac{\theta }{2}+\cos^{2}\alpha_{i}},$$

showing that the deviation angle varies with the folding state. Therefore, the geometric transformation of the limb is not a simple linear deployment but a coupled change in axial length, curvature, and cross-sectional closure. In the proposed design, this orientation change is bounded by the circumferential closure condition. The accumulated deviation must allow the lateral edges of the origami sheet to meet in the folded state, thereby forming the cylindrical terrestrial morphology. At the same time, the same sheet must return to a planar or low-curvature shape in the unfolded state for aquatic paddling. This relationship defines the feasible design space for the sector angle and the number of circumferential modules.

### 3.2. Geometric Transformation Principle and Morphological Evolution

The morphing limb has two functional limit states governed by the folding dihedral angle θ. In State I, corresponding to terrestrial locomotion, the structure approaches the folded configuration. As θ decreases, the origami sheet curls toward a closed cylindrical cross-section. The closed geometry increases structural depth and distributes external contact loads around the circumference. This configuration is therefore used during walking, crawling, and rolling, where the limb must support the body and withstand ground reaction forces.

**Figure 6:**
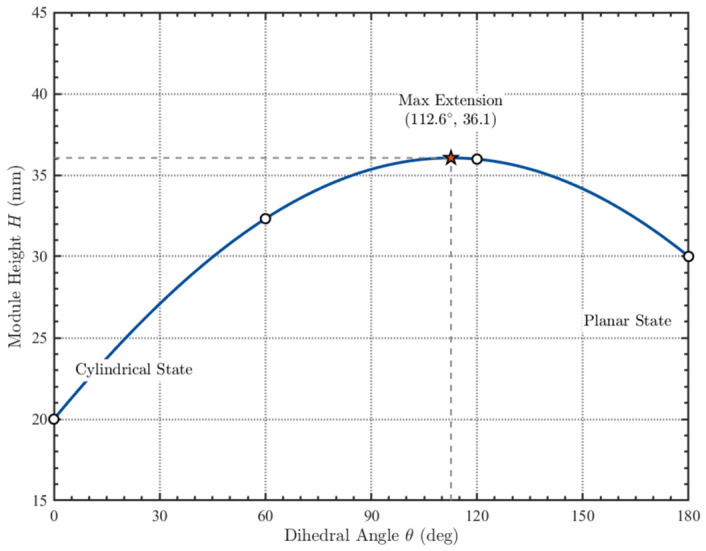
Theoretical variation in the origami module height (*H*) with respect to the dihedral angle (θ). The curve is calculated based on a single-layer configuration (m=1) with geometric parameters h=15 mm and $t_{1}=10$ mm. The marking points indicate the key geometric states (Cylindrical, Max Extension, and Planar), demonstrating the non-linear kinematic evolution of the limb’s axial length.

**Figure 7:**
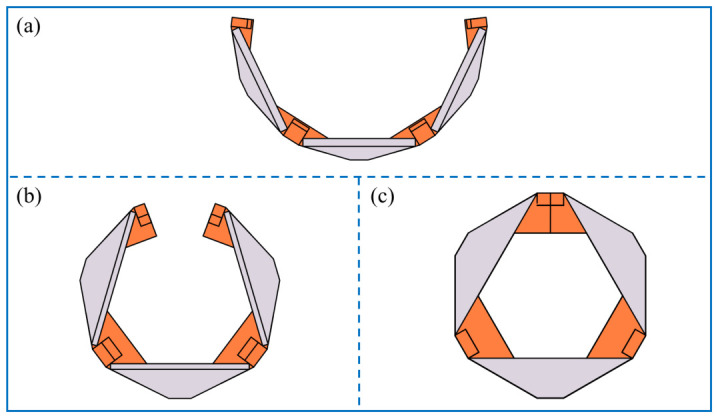
Top-down projection views of the origami structure at different dihedral angles (θ). (**a**) The unfolded configuration at $\theta =120^{\circ }$, showing a low-curvature, paddle-like profile. (**b**) The intermediate transition state at $\theta =60^{\circ }$. (**c**) The fully folded cylindrical state at $\theta =0^{\circ }$, forming a closed load-bearing tube.

In State II, corresponding to aquatic locomotion, the structure unfolds toward a planar configuration. As θ increases, the macroscopic curvature decreases and the projected area increases. The unfolded structure functions as a paddle surface during hip-driven oscillation. In this state, the limb does not primarily rely on column stiffness; instead, it uses its surface area to interact with water and potentially contribute to drag-based propulsion. In addition to the change in cross-sectional curvature, the axial height of the module varies nonlinearly with the dihedral angle, as shown in Figure 6. The module first extends with increasing θ, reaches its maximum height near $\theta =112.6^{\circ }$, and then gradually approaches the planar limit state. The corresponding evolution of the cross-sectional morphology is illustrated in Figure 7. At $\theta =120^{\circ }$, the structure exhibits a low-curvature profile that approximates a planar paddle. At $\theta =60^{\circ }$, the sheet begins to curl, and the morphology transitions from a surface-dominated shape to a volume-forming shape. At $\theta =0^{\circ }$, the lateral edges close into a circular configuration, producing the cylindrical state. This evolution shows how one continuous folding variable can switch the limb between two mechanically distinct functions.

### 3.3. Projected Area Analysis

The potential aquatic paddling capability of the unfolded limb is closely related to the projected area normal to the stroke direction. During drag-based paddling, larger projected area provides a geometric basis for potential drag-based fluid interaction under comparable stroke conditions. The folded cylindrical state has a smaller projected area and is used for terrestrial locomotion, whereas the unfolded state increases the span of the limb and is used for swimming. For a sheet with an unfolded width *W* and length *L*, the theoretical projected area ratio between the aquatic and terrestrial modes can be qualitatively expressed as shown:

(7) $$\frac{A_{\mathrm{aquatic}}}{A_{\mathrm{terrestrial}}}=\frac{W\cdot H_{\mathrm{aquatic}}}{D_{\mathrm{cyl}}\cdot H_{\mathrm{terrestrial}}}=\frac{W\cdot h}{D_{\mathrm{cyl}}\cdot t_{1}}\approx 4.42,$$

where $D_{cyl}$ is the effective diameter of the folded column, *h* is the slant height, and $t_{1}$ represents the effective folded thickness parameter. The approximate area ratio indicates that the proposed limb can substantially change the fluid-interaction area through geometric transformation. This variable-area behavior provides the mechanical basis for using one limb as both a compact terrestrial support and an expanded aquatic paddle.

## 4. Prototype Fabrication and Locomotion Experiments

A physical prototype was fabricated to qualitatively evaluate whether the proposed origami limb can realize the designed shape transformation and support multimodal amphibious locomotion. The prototype demonstrations focused on three aspects: reversible morphological transformation within the tested operating range, terrestrial locomotion using the cylindrical state, and aquatic locomotion using the planar state. The objective of these prototype tests was to evaluate the feasibility of the structural concept and to identify practical issues that should be addressed in subsequent quantitative optimization.

### 4.1. Prototype Fabrication

The origami limb was fabricated using an integrated additive-manufacturing process with two materials. Rigid panels were printed using polylactic acid (PLA), and compliant hinges were printed using thermoplastic polyurethane (TPU) with 95A hardness. The hinge regions were designed as thin flexible sheets with a thickness of 0.4 mm and a width of 0.75 mm. This material distribution allows the panels to remain approximately rigid during folding while the hinge regions provide rotational compliance. The prototype was printed using a Bambu H2D system. The rigid-panel–flexible-hinge architecture reduces the need for discrete mechanical pin joints, simplifies assembly, and enables compact fabrication of the origami module.

**Figure 8:**
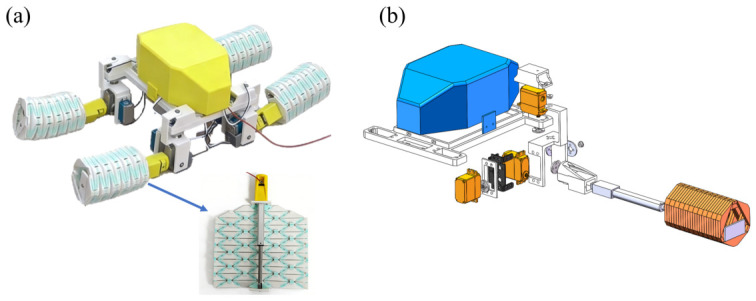
Prototype fabrication and actuation architecture of the origami-inspired amphibious robot. (**a**) Assembled prototype of the quadruped robot. (**b**) Local exploded view of the limb actuation and push-rod mechanism, showing the connection between the LA-T8 linear actuator, the push-rod transmission path, the hip-joint structure, and the origami limb module.

The assembled robot consists of a central chassis and four modular origami limbs. Each limb contains a reconfiguration actuator for folding and unfolding and a hip servo for swing motion. The linear actuator determines the geometric state of the origami limb, while the hip servo provides the periodic motion required for walking, crawling, rolling, and paddling. The selected components and parameters are listed in Table 2. This modular layout allows each limb to be tested as an independent morphing unit while also enabling coordinated quadruped locomotion at the robot level. The assembled prototype and a local exploded view of the limb actuation module are shown in Figure 8a,b, respectively.

The reconfiguration mechanism was implemented as an internal push-rod transmission. As illustrated in Figure 8b, the LA-T8 linear actuator provides an axial displacement along the limb module, and this displacement is transmitted through the push rod to the origami panel assembly. Retraction of the actuator drives the panels toward the folded cylindrical configuration, whereas extension drives the panels toward the unfolded paddle-like configuration. The hip servo is mechanically separated from this reconfiguration path and is responsible for the swing or rolling motion of the whole limb after the target morphology has been reached. Therefore, the linear actuator and the hip servo play different roles: the former controls morphological reconfiguration, while the latter generates limb motion for locomotion.

The control system was implemented using an STM32-based open-loop state sequence. The reconfiguration actuator and the hip servos were commanded sequentially rather than simultaneously. During mode switching, the linear actuator first drove the origami limb to the desired cylindrical or paddle-like configuration. After the reconfiguration process was completed, the hip servos generated the corresponding movements for walking, crawling, rolling, or paddling. Before water entry, the robot therefore followed a transform-then-enter sequence: the limbs were unfolded on land and then used for hip-driven paddling after entering water. This controller should be interpreted as a prototype-level open-loop sequence rather than a closed-loop gait optimizer.

For the water-entry and swimming demonstrations, the joint drives used waterproof servos, while the STM32 controller, battery, communication module, cable connections, and reconfiguration actuator were protected only for short-duration prototype tests. The water experiments therefore demonstrate short-duration amphibious operation of the prototype, but they should not be interpreted as validation of long-term underwater sealing reliability. A systematic waterproof enclosure, cable-feedthrough design, and long-term water-exposure test will be required before outdoor deployment.

### 4.2. Morphological Transformation Verification

The morphological transformation experiment was first conducted to qualitatively evaluate the reconfiguration capability of a single limb and the assembled robot. During the test, the robot was placed in a prone posture to reduce disturbances caused by body motion. The internal push-rod mechanism then drove the origami limb from the folded cylindrical state to the unfolded planar state. The reverse process was also tested to evaluate whether the limb could return to the load-bearing configuration without manual adjustment.

The transformation was observed to be continuous and reversible within the demonstrated operating range of the prototype. The limb changed from a closed cylindrical configuration with a diameter of approximately 78 mm to a planar paddle configuration with a span of approximately 228 mm. The full transition required approximately 30 s under the selected actuator speed. This relatively long transition time is mainly constrained by the rated speed of the LA-T8 linear actuator, the required transmission stroke, and frictional or damping effects in the TPU hinge regions. The slow self-locking actuator was selected to ensure stable and controllable proof-of-concept transformation rather than to optimize rapid mode switching. The current prototype is therefore not intended as a fast-response field robot for highly dynamic shoreline environments. Future designs may reduce the transition time by using faster actuators, shortening the transmission stroke, optimizing hinge geometry, reducing friction, introducing multi-point synchronized actuation, or adding mechanical locking. The transformation did not rely on uncontrolled snap-through motion; instead, the geometry changed gradually under actuator input. This behavior is consistent with the deterministic folding path described by the kinematic model and is suitable for amphibious mode switching, where the timing and configuration of the limb must be controlled.

### 4.3. Multimodal Locomotion Tests

The dynamic processes of limb reconfiguration, walking, crawling, rolling, water entry, and underwater locomotion are provided in Supplementary Video S1. These experiments were designed as qualitative proof-of-concept demonstrations rather than exhaustive quantitative performance measurements. In terrestrial tests, the limbs were maintained in the cylindrical state. In aquatic tests, the limbs were unfolded into the planar state. The primary objective of this stage was to evaluate whether the same structural limb could serve both land and water functions.

#### 4.3.1. Terrestrial Locomotion (Walk, Crawl, and Roll)

In terrestrial locomotion, the folded cylindrical structure was used as the ground-contact element. The closed cross-section allowed the origami limb to support the robot chassis during stance. Walking tests were conducted on gravel-covered grass and on a rubber running track. The robot used a sequential gait in which one limb moved while the remaining limbs supported the body. This gait reduces the risk of body instability and provides sufficient contact time for the cylindrical limbs on uneven surfaces.

A prone crawling mode was also tested. In this mode, the chassis was lowered closer to the ground, reducing the height of the center of mass and increasing contact stability. The crawling test qualitatively showed that the folded origami limbs could provide traction in a low-profile configuration. In addition, the circular cross-section of the folded limb was used to implement a rolling mode. When the hip joints rotated continuously, the cylindrical limbs functioned as wheel-like elements on flat ground. The rolling mode qualitatively demonstrated that the folded morphology can provide both legged support and wheel-like contact, depending on the commanded hip motion.

#### 4.3.2. Amphibious Transition and Swimming

The amphibious transition experiment was conducted using a transform-then-enter sequence. Before entering water, the robot first unfolded its limbs from the cylindrical state to the planar state on land. This operation was completed before water entry so that the reconfiguration actuator did not need to operate during direct water contact or during the unstable transition at the shoreline. After the reconfiguration was completed, the robot moved down a sloped bank in a prone crawling posture. The prone posture lowered the body height and helped the robot maintain contact with the ground during the descent. This procedure avoided changing the limb morphology during the mechanically unstable water-entry phase and reduced the risk of incomplete deployment or unexpected contact with the ground. The unfolded limbs also provided a larger contact area during the approach to the water. Therefore, the transition process was arranged as a simple and conservative operating sequence rather than a simultaneous reconfiguration-and-locomotion maneuver.

After entering water, the robot used the planar limbs as paddle-like structures. Forward motion was generated by reciprocating hip-joint oscillation. During the power stroke, the wide surface of each unfolded limb was oriented to increase the projected area normal to the stroke direction. During the return stroke, the limb was driven back by the same hip joint without using an additional feathering mechanism. The motion was kept simple because this test focused on whether the unfolded limb could work as a paddle. Although this simple motion does not actively change the blade angle between the power and return strokes, it reduces the number of actuators and is sufficient for demonstrating the basic paddling function of the unfolded limb. Therefore, the aquatic motion was produced by the combined effect of the unfolded limb geometry and the periodic hip-joint swing. The swimming experiment showed that the unfolded origami limbs could interact with water as paddle surfaces and produce observable forward motion, supporting the feasibility of the variable-area concept at the prototype scale. This experiment was therefore treated as a qualitative demonstration of the structural concept. Future work will measure swimming speed, thrust, stroke efficiency, and energy consumption.

**Figure 9:**
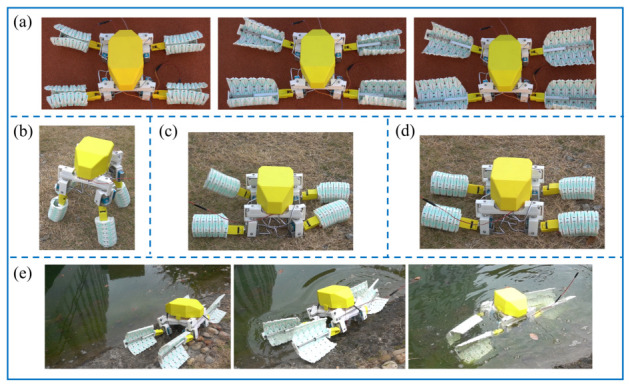
Representative snapshots of the multimodal locomotion experiments extracted from Supplementary Video S1. (**a**) Origami limb reconfiguration. (**b**) Terrestrial walking. (**c**) Terrestrial crawling. (**d**) Rolling locomotion. (**e**) Water entry and underwater locomotion using the unfolded paddle-like limbs.

The representative snapshots corresponding to these demonstrations are summarized in Figure 9. These images follow the same test sequence described above, including limb reconfiguration, terrestrial walking, crawling, rolling, water entry, and underwater locomotion.

## 5. Conclusions

This study presented a bioinspired origami morphing limb for amphibious robots by integrating terrestrial support and aquatic paddling into a single reconfigurable structure. Based on a modified Yoshimura pattern, the limb transforms between two configurations: a folded cylindrical configuration for load-bearing contact and rolling locomotion, and an unfolded paddle-like configuration with an enlarged projected area for drag-based aquatic interaction. This morphology-based strategy reduces the need for separate land and water locomotion modules and provides a compact structural route for amphibious robots operating across land–water transition environments.

To improve transformation reliability, a vertex-splitting thick-panel topology was introduced to suppress the bifurcation ambiguity of the ideal zero-thickness Yoshimura pattern. Finite panel thickness, hinge offsets, and geometric interference constraints guide the structure toward a deterministic single-degree-of-freedom folding path. A screw-theory-based kinematic model was further established to describe the relationship between the active driving angle and the passive folding angle, while geometric analysis connected the folding state with circumferential closure and projected-area variation.

A quadruped amphibious robot prototype was fabricated using rigid PLA panels and flexible TPU hinges. Prototype-level observations qualitatively demonstrated reversible transformation within the tested operating range, as well as walking, crawling, rolling, water-entry, and underwater locomotion. These results support the feasibility of using one origami morphing limb for both terrestrial and aquatic functions. However, the present work remains a proof-of-concept demonstration rather than a complete quantitative performance validation. Future work will focus on measuring locomotion speed, thrust, support stiffness, payload capacity, energy consumption, and transition time; improving actuation speed and hydrodynamic efficiency; and evaluating hinge fatigue, waterproofing reliability, and long-term operation under repeated amphibious use.

## Figures and Tables

**Table 1:** Qualitative comparison with representative amphibious and morphing robotic platforms.

Representative Work	Main Strategy and Validation	Relation to the Present Work
Self-rotating paddle-wheel robot [5]	Paddle-wheel locomotion on land and water; mechatronic prototype tests.	Uses a dedicated wheel/paddle module rather than a morphing support–paddle limb.
Adaptive morphogenesis robot [10]	Turtle-inspired soft-rigid limbs; multi-environment locomotion and cost-of-transport tests.	Shows system-level adaptive morphogenesis; this work studies a thick-panel origami limb.
Bioinspired amphibious origami robot [11]	Origami body for multimodal land–water locomotion; prototype demonstrations.	Uses whole-body origami locomotion; this work focuses on a Yoshimura limb module.
Wireless amphibious origami millirobot [23]	Magnetic Kresling origami body; small-scale rolling, flipping, and fluidic locomotion.	Achieves millimeter-scale wireless motion; this work targets a larger PLA–TPU quadruped limb.
Soft amphibious robot [15]	Pneumatic soft actuators for terrestrial and underwater locomotion; quantitative tests.	Emphasizes soft compliance; this work emphasizes a finite-thickness load-bearing origami state.
Multianimal-inspired soft robot [12]	3D-printed origami actuators integrated with soft structures; amphibious demonstrations.	Uses origami actuators in soft robots; this work studies deterministic support–paddle switching.
This work	Vertex-split thick-panel Yoshimura limb; qualitative demonstrations and Supplementary Video S1.	Investigates limb-level structural integration without claiming quantitative performance superiority.

**Table 2:** Key component selection and parameter specifications of the robot prototype.

Subsystem	Component Name	Key Parameter Specifications
Main Control Unit	STM32F103C8T6	Cortex-M3 core, 72 MHz clock frequency, 64 KB Flash
Joint Drive	S80 Brushless Waterproof Servo	Torque: 60 kg·cm, voltage: 7.4 V, material: stainless steel gears
Wheel Drive	Continuous Rotation Servo	Speed: 60 rpm, torque: 60 kg·cm, dead band: 4 µs
Reconfiguration Drive	LA-T8 Micro Linear Actuator	Stroke: 100 mm, thrust: 60 N, speed: 8 mm/s, self-locking force: 80 N
Power System	2S Lithium Polymer Battery	Capacity: 2200 mAh, discharge rate: 35C
Basic Communication	HC-04 Bluetooth Module	Frequency band: 2.4 GHz, baud rate: 9600 bps
Manufacturing Materials	PLA/TPU 95A	Flexural modulus: 2850 MPa (PLA), elongation at break: 450% (TPU)

## Data Availability

The data presented in this study are available on request from the corresponding author.

## Supplementary Materials

The following supporting information can be downloaded at: https://www.mdpi.com/article/10.3390/biomimetics11070502/s1, Video S1: Dynamic demonstration of the origami morphing limb, including: (i) reconfiguration, (ii) walking, (iii) crawling, (iv) rolling, and (v) underwater locomotion.
